# Miscellaneous

**Published:** 1890-12

**Authors:** 


### MISCELLANEOUS.

### THE FREAKS OF FORTUNE.

A correspondent of the New York *Sun* has furnished some interesting facts regarding the personal history of Mr. James Treadwell, the quicksilver millionaire of California, from which it appears that some thirty years ago Mr. Treadwell left New Brunswick to seek his fortune in the far west. Among his more intimate California friends were a young married couple. The husband was a native of New Brunswick—a fact which will probably account in a great measure for the warmness of the friendship existing between them. Husband and wife were ardent believers in Spiritualism, and were devotedly attached to each other. Mr. Treadwell had little faith in their Spiritualism, and did not hesitate to make known his skepticism. But subsequent events caused a complete change in his opinion. Some years ago Mrs. B. became ill and died. Though removed from her husband in the flesh, he claims that she held frequent communions with him in spirit. During one of these conferences, she expressed a desire to meet Mr. Treadwell, intimating that she had a communication of importance that she wished to make to him. After some coaxing from the husband of the departed woman, Mr. Treadwell consented to meet the spirit. The form appeared to him at the hour and place indicated, and, through the medium of the husband, informed Mr. Treadwell that if he sought in a certain quarter he would find a rich bed of quicksilver.

At first he had little faith in the announcement, but when it was repeated twice he determined to investigate. He had little trouble in locating the spot of land where the spirit had indicated the quicksilver deposit lay, Excavations were begun, resulting in quicksilver being found in immense quantities. Mr. Treadwell pressed upon the husband of the departed spirit to accept a share in his great riches, but he strenuously refused, declaring that he had ample to live upon until he should join his wife in the spirit world. A year ago death visited him, and his desires in this direction were realized. The mine still continues to be worked, and is yielding fabulous wealth to its possessor.

---

Hair-pin Cushion.—This handy little article is very pretty as well as useful to hang on or near the dressing table. It is so simple that a child could make it, and it is inexpensive. Cut a strip of silvered or gilt perforated cardboard 9 in. long and 6 in. wide. Paste a pretty scrap-picture on it or work a simple design in it with bright-colored wool or embroidery silk. Bind the edge of the cardboard with narrow ribbon. Sew the strip of cardboard together so as to form a cylinder, then crochet the circular pieces to fit in the ends. These should be in loose crochet, tufted or looped. Sew one of these circles in place, then fill the cylinder with hair or wool—hair is preferable. Sew on the other end, finish with a ruching made of the ribbon to hide the place where the cushion is joined. Tack ribbon at each end with a small bow, to hang the cushion by.

### THINGS WELL TO KNOW AND DO.

Hot sunshine will remove scorch.

The best liquid for cleaning old brass is a solution of oxalic acid.

Kerosene applied to unused stoves will keep them from rusting.

A damp cloth dipped in common soda will brighten tinware easily.

To clean knives ; cut a small potato, dip it in brick dust and rub them.

Grease may be removed from silk by applying magnesia to the wrong side.

New iron should be gradually heated at first, it will not be so likely to crack.

Paint splashes may be removed from window panes by a very hot solution of soda, using a soft flannel.

Mildewed linen may be restored by soaping the spots, and while wet covering them with powdered chalk.

### A FAR OFF STAR.

It is difficult to conceive that the beautiful dog star is a globe much larger than our sun, yet it is a fact that Sirius is a sun many times more mighty than our own. This spendid star, which, even in our most powerful telescopes, appears as a mere point of light, is in reality a globe emitting so enormous a quantity of light and heat that were it to take the place of our own sun, every creature on this earth would be consumed by its burning rays.

Sirius shining with far greater lustre than any other star, it was natural that astronomers should have regarded this as being the nearest of all the "fixed" stars ; but recent investigation on the distances of the stars has shown that the nearest to us is Alpha Centauri, a star belonging to the southern latitude, though it is probable that Sirius is about fourth on the list in the order of distance. For, though there are about fifteen or twenty stars whose distances have been conjectured, the astronomer knows that in reality, all of them, save three or four, lie at distances too great to be measured by any instruments we have at present.

Astronomers agree in fixing the distance of the nearest fixed star at 22,000,000,000,000 miles, and it is certain that the distance of Sirius is more than three and less than six times that of Alpha Centauri, most likely about five times, so that we are probably not far from the truth if we set the distance of Sirius at about 100,000,000,000,000 miles. What a vast distance is this that separates us from that star ! Words and figures themselves fail to convey to our minds any adequate idea of its true character.

To take a common example of illustrating such enormous distances : It is calculated that the ball from an Armstrong 100-pounder quits the gun with the speed of about four hundred yards per second. Now, if this velocity could be kept up, it would require no fewer than 100,000,000 years before the ball could reach Sirius.

Palpitation of the Heart.—An excessive palpitation of the heart can always be arrested by bending double, with the head down, and the arms pendant, so as to produce a temporary congestion of the upper part of the body. If the respiratory movements be suspended during this action, the effect will be only the more rapid.

### DRUGGED BAKING POWDERS.

### HOW THE PRESENCE OF AMMONIA IN A POWDER MAY BE DETECTED.

Ammonia and alum are the most common adulterants used in the manufacture of baking powders. The Government report shows that a large percentage of the baking powders on the market contain either one or the other, or both these pernicious drugs. Ammonia particularly is in very general use. So great has the demand for it increased with the increased use of baking powders, that an ammonia trust has been formed and the price of the drug has gone up. Such baking powder companies as are not share holders in the trust company have suffered a material decrease in profits as a consequence.

This wholesale use, in an article of daily food, of one of the most insidious and injurious poisons is simply criminal. Slow ammonia poisoning produces diseases of the stomach and is particularly injurious to the complexion. The presence of ammonia in a baking powder, however, can be very easily detected. Mix one heaping teaspoonful of baking powder with one teaspoonful of water in a tin cup; boil thoroughly for a few moments, stir to prevent burning, and if ammonia is present you can smell it in the rising steam. As baking powder, when first thrown into the water, will effervesce, care should be taken to not mistake bubbling for boiling. When a baking powder has not a list of its ingredients printed on its label this test should always be applied.

Ammonia, in a measure, takes the place of the expensive ingredients of baking powders. Where sales are large the profits which this adulteration yields are enormous and some of the best known manufacturing firms whose standing would seem a guarantee against fraud are engaged in it. Even some of those concerns which advertise the most extensively and whose advertisements ring with the "purity" of the article, are among the worst offenders.

---

How to Clean Furs.—Ermine and minever are best cleaned with soft flannel. Rub the fur well against the grain; then dip the flannel into common flour and rub the fur until clean; shake the fur, and again rub it with a fresh piece of soft, clean, new flannel till the flour is out. By this process the color of the ermine is preserved and the lining need not be removed. Sable, chinchilla, squirrel, etc., are cleaned with new bran, which must be warmed very carefully in a pan, but not burnt; therefore, while warming, stir it frequently. Rub the warm bran into the fur for some time, shake it and brush until free from bran. The fur will clean better if the stuffing and lining are removed and the article laid as straight and flat as possible on a table or board. Well brush the fur before it is cleaned, and if there are any moth-eaten parts they must be removed and replaced with new pieces. The following method is said to be adopted in Russia: Some rye flour is put into a pan upon the stove and heated, being stirred constantly with the hand as long as the heat can be borne; then the flour is spread all over the fur and rubbed in well. It is then brushed gently with a very clean brush, or beaten softly till all the flour is removed. It is claimed that this method will make the fur appear almost like new.

HERBA VITA, a simple and natural remedy, advertised in these columns, will accomplish all that quinine is capable of, and leave no baneful or injurious effects in the system. It is not only prophylactical and healing, but thoroughly cleansing to the vital energies. Try it at a trifling cost and spare the doctor his long, troublesome bill. Do not mistake this for any of the rank herb teas so largely advertised and blame us for a failure. It is not a tea, but a pure decoction of leaves and roots of wonderful curative properties, that will not harm an infant.

### WILL YOU DO IT, BRETHREN ?

A society has been organized at Hannibal, Mo., under the suggestive name of "THE BROTHERHOOD OF MORALISTS," one of its aims being to eradicate from the mind a belief in *spirit manifestations*, as of the same piece with witchcraft. Perhaps they may succeed, but not with those who have the time, patience and courage to search for the *truth*, and to declare it.

---

BABY LAND and OUR LITTLE MEN AND WOMEN are growing in the favor of the little folks as they deserve to, for their efforts to please them. D. Lothrop & Co., Boston.

---

NEW YORK, NOV. 11, 1890.

HERBA VITA REMEDY CO.,

I feel it a duty to inform you of the remarkable cure which your preparation effected in my case. I had been afflicted for a long time by a complication of disorders for which I vainly sought relief. My appetite failed me, my food did not seem to digest, and my face was bloated out of all natural proportions. A friend, seeing my condition, advised me to try HERBA VITA, of which I had never previously heard. I procured a package and experienced such relief from it that I continued the treatment through several weeks, till now, I find my health fully restored without a trace of the troublesome disorders left, and for the first time in months, I have a clear, natural complexion and sleep with the soundness of a babe. I am confident I owe my restoration to health to HERBA VITA, and for this you have my thanks.

CARRIE G. HADDEN.

---

### THE FAIR THEOSOPHIST.

She cares not for the worldly things
  That entertain the rest of us ;
She soars, on spiritual wings,
  Far, far above the rest of us.
She smiles, but yet declines to mix,
  With diffident apology,
In mothers' meetings, politics,
  Lawn tennis, or theology.

So elevated is her mind,
  This life is far too rough for her,
And orthodoxy can not find
  A heaven good enough for her.
She asks, and we forgive the sin,
  In charming femininity,
She asks to be a Chela in
  Thibet, or the vicinity.

—*Pall Mall Gazette.*

